# High Prevalence and Factors Associated With the Distribution of the Integron *intI1* and *intI2* Genes in Scottish Cattle Herds

**DOI:** 10.3389/fvets.2021.755833

**Published:** 2021-10-29

**Authors:** Cristina Fernández Rivas, Thibaud Porphyre, Margo E. Chase-Topping, Charles W. Knapp, Helen Williamson, Olivier Barraud, Sue C. Tongue, Nuno Silva, Carol Currie, Derek T. Elsby, Deborah V. Hoyle

**Affiliations:** ^1^The Roslin Institute, Royal (Dick) School of Veterinary Studies, University of Edinburgh, Easter Bush, Scotland, United Kingdom; ^2^Laboratoire de Biométrie et Biologie Évolutive, UMR5558, CNRS, VetAgro Sup, Université de Lyon, Villeurbanne Cedex, France; ^3^Centre for Water, Environment, Sustainability and Public Health, Department of Civil & Environmental Engineering, University of Strathclyde, Glasgow, United Kingdom; ^4^INSERM, CHU Limoges, UMR1092, Université de Limoges, Limoges, France; ^5^Epidemiology Research Unit, Scotland's Rural College (SRUC), An Lòchran, Inverness Campus, Inverness, United Kingdom; ^6^Moredun Research Institute, Edinburgh, United Kingdom; ^7^Environmental Research Institute, University of the Highlands and Islands, Thurso, United Kingdom

**Keywords:** integron, cattle, Scotland, prevalence, risk factors, epidemiology, antimicrobial resistance (AMR)

## Abstract

Integrons are genetic elements that capture and express antimicrobial resistance genes within arrays, facilitating horizontal spread of multiple drug resistance in a range of bacterial species. The aim of this study was to estimate prevalence for class 1, 2, and 3 integrons in Scottish cattle and examine whether spatial, seasonal or herd management factors influenced integron herd status. We used fecal samples collected from 108 Scottish cattle herds in a national, cross-sectional survey between 2014 and 2015, and screened fecal DNA extracts by multiplex PCR for the integrase genes *intI1, intI2*, and *intI3*. Herd-level prevalence was estimated [95% confidence interval (CI)] for *intI1* as 76.9% (67.8–84.0%) and *intI2* as 82.4% (73.9–88.6%). We did not detect *intI3* in any of the herd samples tested. A regional effect was observed for *intI1*, highest in the North East (OR 11.5, 95% CI: 1.0–130.9, *P* = 0.05) and South East (OR 8.7, 95% CI: 1.1–20.9, *P* = 0.04), lowest in the Highlands. A generalized linear mixed model was used to test for potential associations between herd status and cattle management, soil type and regional livestock density variables. Within the final multivariable model, factors associated with herd positivity for *intI1* included spring season of the year (OR 6.3, 95% CI: 1.1–36.4, *P* = 0.04) and watering cattle from a natural spring source (OR 4.4, 95% CI: 1.3–14.8, *P* = 0.017), and cattle being housed at the time of sampling for *intI2* (OR 75.0, 95% CI: 10.4–540.5, *P* < 0.001). This study provides baseline estimates for integron prevalence in Scottish cattle and identifies factors that may be associated with carriage that warrant future investigation.

## Introduction

Antimicrobial resistance (AMR) is a serious global health problem that prevents the effective treatment of bacterial infections in humans and animals worldwide and requires a One Health approach (1, 2). Widespread use of antimicrobial drugs within clinical health settings and the livestock industry drive resistance development, and both contribute to environmental contamination with resistant bacterial populations (3, 4). Antimicrobial resistance genes (ARG) are disseminated through two main routes, the vertical transmission of chromosomally encoded resistance genes to daughter cells and the horizontal transfer of genes between bacterial cells facilitated by mobile genetic elements, such as plasmids, transposons, and phage DNA (5).

Integrons are genetic elements that function as gene capture and expression systems, integrating single or multiple gene cassettes at the *attI* recombination site by means of the integrase, *intI*, gene (6, 7). Captured genes are expressed through a single *Pc* promoter located within the 5′ conserved segment of the integron. Carriage of several stacked gene cassettes within integrons is common, enabling the linked dissemination of multiple genes within arrays by a single element (6, 8). Integrons lack a transposase gene and are therefore not self-mobile, however their horizontal movement between genomes is facilitated when integrons are hosted by transposons or conjugative plasmids. There are several classes of integron found in many bacterial species worldwide, from diverse human, animal and environmental origins, however only classes 1 to 3 are thought to be of clinical relevance within human healthcare or livestock settings (9).

Class 1 integrons are particularly associated with multiple drug resistance within *Enterobacteriaceae* (10–13), due to their ability to incorporate and stack a wide repertoire of gene cassettes within arrays (14–16). For this reason, the *intI1* integrase gene has been proposed as a proxy measure for general surveillance of ARGs within clinical settings (17), livestock populations and environmental habitats (18–20). Monitoring *intI1* prevalence provides additional information on the potential for transmission of multiple drug resistance traits, complementing the surveillance of resistance to specific antimicrobial drug classes (21). In contrast, class 2 integrons tend to carry only a limited range of gene cassettes, generally those encoding resistance to trimethoprim, streptothricin and streptomycin, due to the presence of a defective mutation within the *intI2* gene preventing acquisition of further cassettes (22). Class 2 integrons have been isolated from commensal and pathogenic intestinal bacteria of humans and animals, but are less commonly detected in the environment than class 1 (9, 15). Class 3 integrons are relatively rare, with most reports relating to the identification of *intI3* in human clinical strains (23), or in bacteria isolated from hospital waste effluents and sewage (24, 25).

Many integrons also carry genes conferring resistance to quaternary ammonium compounds (QAC) (7, 8), such as the *qacE*Δ*1* gene, found within the conserved 3′ region of a subgroup termed “clinical” class 1 integrons (26). QACs are present within a variety of detergents and disinfectants, hence biocide use within human health and agricultural settings may directly select for integron carriage (27–29). Further, integrons are frequently linked with genes conferring resistance to heavy metals, through their physical proximity within hosting transposons and plasmids to genes associated with metal resistance or metabolism (7, 30, 31). The presence of the integrase *intI1* gene within environmental bacterial populations has therefore been proposed as an indicator for the level of anthropogenic pollution within habitats (28, 32–34). Where antimicrobial, biocide, and metal resistance genes co-occur, gene linkage may facilitate acquisition and retention of ARGs within populations in the absence of direct selection pressure (35, 36). This has implications for the reduction of ARGs within livestock reservoirs by limiting therapeutic or prophylactic usage of antimicrobials, since resistant bacterial populations may be maintained irrespective of treatment practices.

The aim of this study was to estimate the prevalence and spatial distribution of the *intI1, intI2*, and *intI3* integron genes by real-time PCR in Scottish cattle herds, using fecal samples collected as part of a national, cross-sectional study of cattle in Great Britain, the “British *E. coli* O157 in Cattle Study” (BECS) (37). We sought to determine whether specific epidemiological or herd management factors influenced carriage of integron elements, as vehicles of dissemination, since this may provide additional insight into how genes spread through or are retained within livestock populations, irrespective of individual ARG type. Using external datasets, we tested the hypotheses that soil integron abundance in Scotland, land type or local livestock density may be associated with herd integron status. The estimation of integron carriage in Scottish cattle herds will provide a baseline for prevalence in this sector, together with an indication of the wider potential for ARG dissemination within agricultural environments in Scotland. These data may inform the existing knowledge base and action plans for the control of AMR in livestock (2, 38).

## Materials and Methods

### Study Population and Herd Demographics

A collection of frozen samples was used for this study, derived from the Scottish survey component of the national, cross-sectional British *E. coli* O157 Cattle Study (BECS) (37). Samples were provided as 6 h fecal pat enrichment cultures in buffered peptone water (BPW), preserved in 15% v/v glycerol and frozen at −80°C. In BECS, individual, fresh fecal pat samples were collected from the cattle group nearest to slaughter, over a thirteen-month period between September 2014 and September 2015. Samples were collected at random from intact, discrete pats present on the ground of the pen or field in which the animals were held. The overall number of herds included in the study and the number of samples taken per herd were based on a previous sampling framework for estimating *E. coli* O157 prevalence in Scotland, designed to estimate prevalence with a sensitivity of 90% and confidence of 96% for an expected herd prevalence level of 20.5% (37). For the current study reported here, the Scottish dataset consisted of 108 herds from which 2,755 individual, fresh fecal pat samples were available (median fecal pat number per herd of 23; range, 7–75).

Herd management data was obtained by questionnaire completed through face-to-face interviews at the sampling visit. Variables included herd and sampling group size, farm type, management, cattle movements, water source, health status, any veterinary medicines administered to the group in the preceding 3 months and the presence of other species on the farm. A summary of the herd data demographics is outlined in Henry et al. (37) and a copy of the questionnaire is available upon request from the BECS study corresponding author.

### DNA Preparation and Sample Pools

DNA was extracted from a 50 μl aliquot of each individual fecal enrichment culture using InstaGene™ matrix (Bio-Rad Laboratories Ltd, Watford, UK), as previously described (39). To confirm success of DNA extraction and the absence of PCR inhibitors in each fecal extract, phocine herpes virus (PhHv) glycoprotein B (40) (gifted by Dr Lesley Allison, Scottish *Escherichia coli* Reference Laboratory, Edinburgh, Scotland) was spiked into the InstaGene™ matrix (Bio-Rad) as an internal inhibition control and verified on an individual DNA extract basis, as previously described (39).

We used a pooling methodology (41, 42) to determine herd status for *intI1, intI2*, and *intI3*. In the current study, for each herd, pools comprising five individual fecal DNA extracts were mixed as 10 μl aliquots of each individual extract, to form a 50 μl pool, on ice. A minimum of two pools were tested for every herd; if either pool was designated positive for the gene target of interest, no further pools were assessed in that herd. However, where the first two pools in a herd were designated negative, subsequent pools comprising the next five fecal DNA extracts were sequentially tested, until either a positive pool was recorded, or all available fecal DNA extracts had been tested for that herd. In cases where fewer than five fecal DNA extracts remained within a herd for a pool, nuclease free water (Qiagen, Crawley, UK) was used to make up the equivalent volume, so that all individual DNA extracts were present as a 1:5 ratio within all pools. It was beyond the scope of this study to test every possible individual fecal DNA extract on a herd basis for *intI1* and *intI2*, or to determine *intI3* herd status by testing every possible pool in a herd, due to resource constraints.

### Bacterial Control Strains

Positive control strains were gifted by Dr Olivier Barraud, University of Limoges (Limoges, France) as follows: *E. coli* DH5α for *intI1* within plasmid pBAD18; *E. coli* JM109 for *intI2* within plasmid pGEM-T Easy and *E. coli* DH5α for *intI3* within plasmid pBAD18 (43). *E.coli* strain K12 MG1655 was used as a negative control. DNA was extracted from the control strains using InstaGene™ matrix (Bio-Rad) and included on each reaction plate. In order to determine gene copy number, plasmids were extracted using the Wizard^®^ Plus SV miniprep DNA purification system (Promega, Southampton, UK) following manufacturer's instructions and DNA measured on a NanoDrop^TM^ 1,000 Spectrophotometer (Thermo Fisher Scientific, US). Integron gene copy number was calculated according to Barraud et al. (43).

### Real-Time PCR Herd-Level Screening for Integron Genes

The presence of the class 1, 2, and 3 integrase genes *intI1, intI2*, and *intI3* was assessed by a multiplex real-time PCR assay developed and validated by Barraud et al. (43, 44). Genes were amplified with the primers and probes as described in Supplementary Table 1. A no template control (NTC) of nuclease-free water (Qiagen, Crawley, UK) and DNA extract of the *E. coli* strain MG1655 were included as negative controls. All samples were run in duplicate and reactions were conducted in a 20 μl volume, consisting of 2 μl of DNA template (pool or control), 10 μl of QuantiTect Multiplex PCR NoROX (Qiagen, Crawley, UK), 0.4 μM of each primer, 0.2 μM of each probe, with nuclease-free water (Qiagen) up to volume. The assay was performed in a Bio-Rad CFX96 Real-Time System, C1000 Touch^TM^ Thermal Cycler with an initial denaturation step of 10 min at 95°C, followed by 40 cycles of 95°C for 30 s and 60°C for 1 min, with a final extension step of 25°C for 2 min. Data was captured using the Bio-Rad CFX Manager 3.1 programme (Bio-Rad, US).

Thresholds were set so that the quantification cycle value of the standard samples produced the least variability and was applied across all plates at a relative fluorescence unit of 300 for FAM-*intI1*, 200 for Texas Red-*intI2* and 150 for Cy5-*intI3*. Pool sample cycle threshold (C_t_) values were read at these values and exported in.xlsx format for analysis within Excel (Microsoft, US). For case definition, a pool sample was recorded positive if the C_t_ value was less than or equal to the mean positive control standard cut-off at a value equivalent to 50 gene copies (*intI1*, C_t_ = 32.13; *intI2*, C_t_ = 34.36; *intI3*, C_t_ = 32.86). Repeat assays were performed on a number of pools as standard and to confirm negative herd status (Supplementary Table 2). A herd was considered positive if at least one of the pools tested in the herd was positive for the target gene of interest. The minimum observed pool C_t_ values were recorded per herd and summarized by the outcome variables found to be of significance in the risk factor modeling.

### Datasets for Land Type, Livestock Densities and Soil Integron Abundance

#### Land Type

Herd holdings were classed according to the Macaulay Land Capability for Agriculture in Scotland (LCA) classification, a system developed to describe the agricultural potential of land, based on the degree of limitation imposed by biophysical properties (45). Classification is made according to climate, soil properties (e.g., depth/stoniness), wetness, erosion risk and slope, as well as variability and vegetation cover. The LCA is a seven class system where class 1 represents land that has the highest potential flexibility of use, whereas class seven land is of very limited agricultural use. LCA and soil composition data in the local region of the herd holding site, including presence of mineral iron podzols, was obtained from the National Soil Map dataset (Version 1.4), James Hutton Institute (46) (https://www.hutton.ac.uk/learning/natural-resource-datasets/soilshutton/soils-maps-scotland/download).

#### Livestock and Holding Density

Potential associations between *intI1* and *intI2* herd status and regional cattle, sheep and pig density and livestock holdings density within the locality of the sampled herd holdings were examined. Cattle and sheep density, as well as cattle and sheep holdings density, were sourced from the 2011 Scottish agricultural census dataset *via* agcensus.edina.ac.uk (47). Data for regional pig density and pig holdings density were computed based on all pig holdings actively moving pigs in both Scotland and the rest of Great Britain between January 2012 and December 2013, and recorded in movement datasets from Scotland (ScotEID) and England and Wales (eAML2), as detailed in Porphyre et al. (48). Of the 15 livestock and holding density pairwise correlations, 12 were significantly correlated (Pearson correlation, *p* < 0.05) (Supplementary Table 3). As a result, a Principal Component Analysis was performed to generate independent variables to use in the risk factor analysis (Supplementary Figures 1–3).

#### Soil Integron Abundance

The abundance and spatial distribution of *intI1* and *intI2* genes in Scottish soils was obtained from the dataset, “Antibiotic resistance genes found in soils across the entire Scottish landscape” by Knapp et al. (49). This dataset comprises ARG abundance, including *intI1* and *intI2*, within whole soil sample DNA extracted from soils held by the National Soils Inventory of Scotland archive (NSIS2). These soil samples were originally collected between 2007 and 2010 from 183 locations across Scotland, using a 20 km square grid sampling framework. Soil *intI1* abundance in the locality of the herds was included as a variable to test for association with individual herd status, as described below.

A kernel smoothing method (50) was used to model density variations of overall cattle, sheep and pig numbers and holding distributions, as well as soil *intI1* abundance, and to interpolate mean estimates between observation events. A weighted kernel intensity ratio method, implemented in the btb package (51) of the statistical software R (version 4.0.5) (52), was used to compute smoothed maps of 5–10 km-wide square cells, implementing an edge-correction for the Scotland/England border and along the coastline. The bandwidth parameter for the kernel functions used to control the degree of smoothing was fixed to 15 km for cattle, sheep and pig densities and holding densities, and to 50 km for soil *intI1* abundance. The spatial distribution of soil *intI1* and *intI2* abundance (Supplementary Figure 4) was plotted using R software, with smoothed distribution for *intI1* (Supplementary Figure 5).

### Statistical and Epidemiological Analysis

#### Prevalence Estimates

Herd-level prevalence was estimated using a method similar to Henry et al. (37). Briefly, a generalized linear mixed model (GLMM) with a logit link fitted with a random herd effect to model extra-binomial variability was performed using Proc Glimmix (SAS version 9.4). Mean estimates and CIs were generated by back transforming from the output on the logit scale. Overall prevalence was estimated separately for each integron *(intI1* and *intI2*). Prevalence estimates were also calculated for different spatial (Animal Health District, AHD) and temporal (season) factors. Herds were categorized into the following six Scottish Animal Health Districts (AHD) according to geographic location: Highland (18 herds), North East (19 herds), Central (16 herds), South West (22 herds), South East (17 herds), and Islands (16 herds). Season was defined as autumn (September–November), winter (December–February), spring (March–May) and summer (June–August).

#### Risk Factor Analysis

Risk factors for the presence of *intI1* and *intI2* in a herd were analyzed using GLMM (Proc Glimmix). The unit of analysis was herd level status (positive/negative). A herd was considered positive if at least one of the pools tested in the herd was positive for the target gene of interest. Generalized linear models were initially carried out on a single variable basis. All the potential risk factors (Supplementary Table 4, *n* = 61) were examined. In order to control for confounding, exploratory data analysis was performed before running the model, including examination of correlations among all variables. When two variables were highly correlated the variables with the best Bayesian information criterion (BIC) was used in the model, although these variables were replaced with correlated variables as part of the model checking procedure. For the livestock density variables we performed a Principal Component Analysis to alleviate the correlations amongst the livestock density data, as previously described. All variables with a *P*-value of <0.25 were retained for the multiple variable analysis. For the multivariable analysis, region (AHD) and season were forced into the model as design factors. A backward elimination approach with swapping (reassessment of previously included or excluded variables) was used. The change in the BIC of the model was monitored as an indicator of improved fit. Variables were added and removed based on significant improvement in the BIC after changes to the model. Two-way interactions were also tested in this manner. Herd was fitted as the sole random effect to the final model to help model the extra-binomial variability. To check for multicollinearity between factors in the final model, correlations were examined for binary and nominal variables. In addition, the stability of the model was checked by systematic removal of variables. Diagnostics were performed and plots of residuals were examined, confirming goodness of fit of the model. Odds ratios and their associated 95% CI were estimated in the final model for factors statistically significantly associated with the presence of *intI1* and *intI2*.

All statistics were performed using SAS (SAS Institute Inc., Cary, NC), unless otherwise specified. A *P*-value of 0.05 was accepted as the level of significance.

## Results

Seventy seven percent of herds (83/108) were positive for the *intI1* gene and 82% of herds (89/108) were positive for the *intI2* gene. Dual detection of both *intI1* and *intI2* in herds was common, with 68% (73/108) of herds positive for both genes, whereas only 8% (9/108) of herds were negative to both *intI1* and *intI2*. Ten herds were positive only to *intI1* and sixteen herds were positive only to *intI2*. The majority of herds designated positive were identified as positive within the first two pool samples tested, in 89% (74/83) and 92% (82/89) of positive herds for *intI1* and *intI2*, respectively; 99% of all positive herds were identified within the first four pools tested for both *intI1* (82/83 herds) and *intI2* (88/89 herds). No response to the *intI3* gene was detected in any of the pools tested.

**Figure 1:**
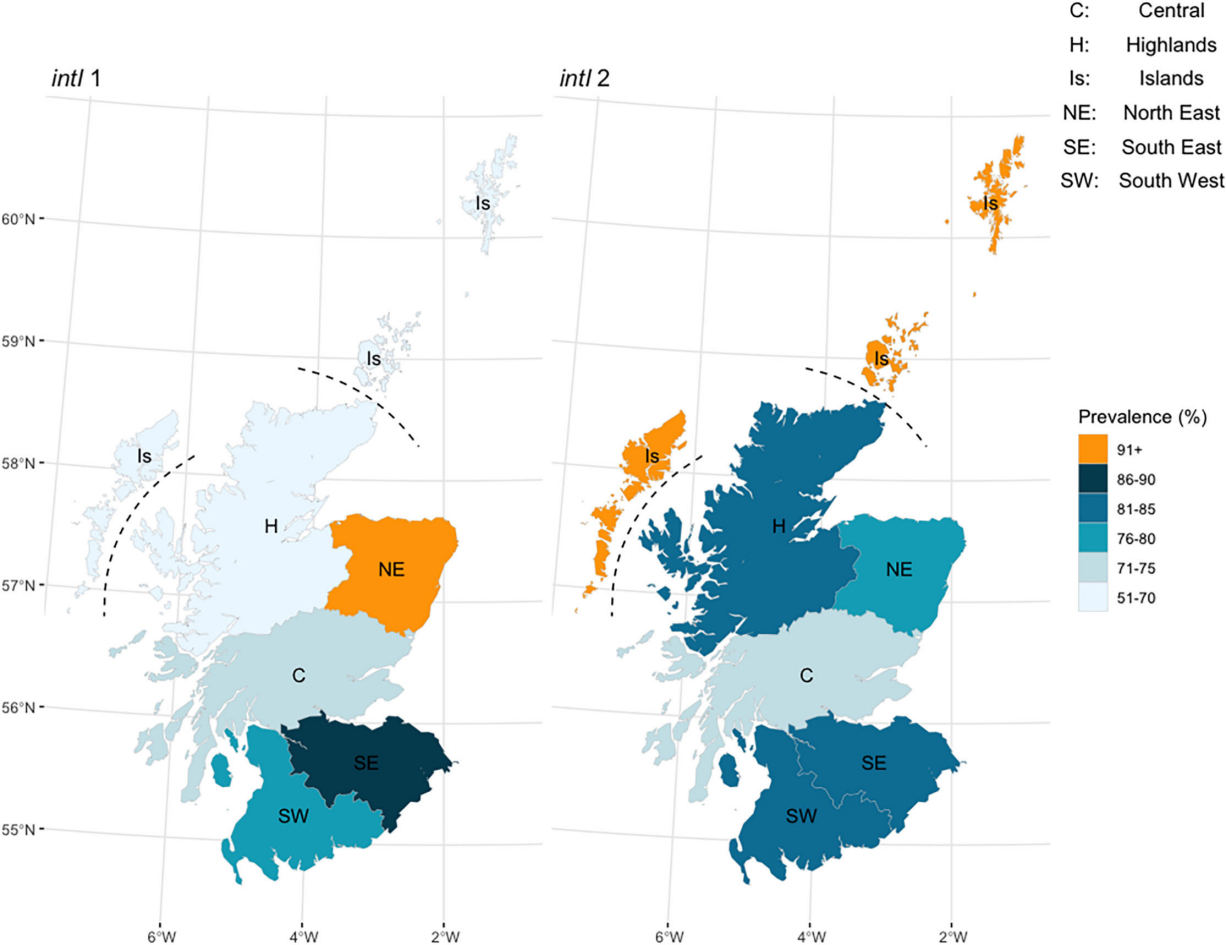
Observed (raw) herd prevalence by Animal Health District region for the integron *intI1* and *intI2* genes in 108 Scottish cattle herds, by real-time PCR.

The observed (raw) prevalence for *intI1* and *intI2* in the study herds was examined by Animal Health District (AHD) to investigate possible geographical variation across Scotland (Figure 1). Observed herd prevalence was highest for *intI1* in the North East and lowest in the Highlands, and for *intI2* was highest on the Islands and lowest in the Central region. A generalized linear mixed model was used to estimate the national cattle herd prevalence for the *intI1* and *intI2* genes in Scotland. The mean herd prevalence, with 95% confidence intervals (CI), was 76.9% (67.8–84.0) for *intI1* and 82.4 % (73.9–88.6) for *intI2* (season and region estimates, Supplementary Table 5).

The univariable analysis results for all 61 potential risk factors for association with *intI1* and *intI2* herd level status are shown in Supplementary Tables 6, 7 with odds ratios (OR) calculated for variables where *P* < 0.25 (Tables 1, 2). At the univariable level, a significant regional effect was observed between Highland and North East regions for *intI1* (*P* = 0.022) and trend toward significance for Highland to South East (*P* = 0.052); no regional variation was observed for *intI2*. A number of herd management factors were associated with herd status. For *intI1* the number of cattle in the herd aged 12–30 months, farms allowing movement of cattle into the herd, the use of water derived from a natural spring source and cattle housed at time of sampling were all significant factors associated with positive herd status, whilst presence of geese on fields was associated with a negative status. For *intI2*, being housed at the time of sampling, the presence of sheep on the farm and spring season of the year were significant risk factors within the univariable model. The herd management questionnaire included data on health status and any treatments administered to the sampled group within 3 months of sampling: no association was observed between these variables and herd status for either *intI1* or *intI2*. None of the land or soil characteristics measured in the locality of a herd, including the land classification (LCA), general soil type or specific soil types were found to be significantly associated with *intI1* or *intI2* individual herd status (Supplementary Tables 6, 7).

**Table 1:** Region, season and herd management variable association with herd *intI1* PCR status, by a univariable generalized linear mixed model, given as odds ratio with corresponding 95% confidence intervals (CI), for variables where *P* < 0.25.

**Variable**	**Coeff^a^**	**SE^a^**	***P***	**Odds ratio (95% CI)**
**Animal Health District**				
Central	0.88	0.77	0.261	2.40 (0.52–11.19)
Highland	–	–	–	–
Island	0.57	0.74	0.450	1.76 (0.40–7.79)
North East	2.67	1.15	0.022	14.40 (1.48–140.0)
South East	1.79	0.91	0.052	6.00 (0.99–36.50)
South West	1.00	0.72	0.168	2.72 (0.65–11.40)
**Season**				
Autumn	1.09	0.67	0.103	2.99 (0.80–11.19)
Winter	0.06	0.63	0.919	1.07 (0.30–3.76)
Spring	1.16	0.71	0.103	3.20 (0.79–13.02)
Summer	-	-	-	-
**Continuous variables**				
Number cattle 12–30 months^b^	0.85	0.34	0.014	2.35 (1.96–4.61)
Total number of cattle^b^	1.03	0.55	0.063	2.80 (0.94–8.30)
Pig Density^b^	0.33	0.25	0.188	1.39 (0.09–2.26)
Cattle Density^c^	0.30	0.12	0.013	1.35 (1.07–1.70)
Cattle Holding Density^c^	0.40	0.20	0.042	1.50 (1.02–2.21)
Sheep Density^b^	1.52	0.73	0.040	4.57 (1.07–19.50)
PC1^d^	0.30	0.14	0.029	1.35 (1.03–1.77)
**Categorical variables**				
Poultry present	0.82	0.60	0.176	2.26 (0.69–7.43)
Cattle brought onto farm	1.28	0.52	0.016	3.59 (1.29–10.02)
Slurry spread	0.58	0.49	0.232	1.80 (0.69–4.70)
Wild geese present	−1.04	0.48	0.032	0.35 (0.14–0.91)
Cattle spring water	1.47	0.52	0.006	4.34 (1.54–12.22)
Farmhouse private water	0.66	0.56	0.240	1.29 (0.64–5.81)
Group housed at sampling	1.11	0.50	0.032	3.02 (1.11–8.26)
Changed feed	0.97	0.67	0.148	2.65 (0.70–9.93)
Changed location	1.09	0.79	0.172	2.96 (0.62–14.16)
Brown soil^e^	0.73	0.50	0.150	2.07 (0.77–5.59)
Mineral podzol^e^	−0.76	0.52	0.150	0.47 (0.17–1.32)

a *Coeff: coefficient; SE: Standard error*.

b *(Log_10_) transformed.*

c *Square root transformed*.

d *PC1 (Principle Component 1) is an index variable created by Principal Components Analysis (Minitab v.18). PC1 explains 52.7% of the variation in the data. Larger values of PC1 represent higher number of holdings and density of cattle, sheep, and pigs*.

e *Land defined as suitable for arable use within James Hutton Land Capability for Agriculture (LCA) classification code (https://www.hutton.ac.uk/learning/exploringscotland/land-capability-agriculture-scotland)*.

**Table 2:** Region, season and herd management variable association with herd *intI2* PCR status, by a univariable generalized linear mixed model, given as odds ratio with corresponding 95% confidence intervals (CI), for variables where *P* < 0.25.

**Variable**	**Coeff^a^**	**SE^a^**	***P***	**Odds ratio (95% CI)**
**Animal Health District**				
Central	–	–	–	–
Highland	−0.88	0.77	0.261	0.42 (0.09–1.94)
Island	−0.31	0.81	0.705	0.73 (0.15–3.71)
North East	1.79	1.19	0.137	6.00 (0.56–64.06)
South East	0.92	0.97	0.346	2.50 (0.37–17.08)
South West	0.13	0.79	0.875	1.13 (0.24–5.46)
**Season**				
Autumn	0.31	0.62	0.617	1.37 (0.39–4.75)
Winter	1.57	0.88	0.076	4.81 (0.84–27.44)
Spring	1.74	0.87	0.049	5.69 (1.01–32.13)
Summer	-	-	-	-
**Continuous variables**				
Pig density^b^	−0.32	0.25	0.214	0.73 (0.44–1.20)
Pig holding density^c^	−0.56	0.32	0.082	0.57 (0.30–1.08)
PC1^d^	−0.21	0.15	0.183	0.81 (0.60–1.10)
**Categorical variables**				
Sheep present	1.20	0.53	0.024	3.33 (1.18–9.45)
Other animals present	−0.63	0.51	0.220	0.53 (0.19–1.47)
Cattle brought onto farm	0.67	0.57	0.243	1.96 (0.63–6.08)
Other livestock brought on	0.83	0.53	0.116	2.30 (0.81–6.53)
Group housed at sampling	3.86	0.73	<0.001	47.40 (11.20–200.60)
Cattle health	−1.38	0.83	0.102	0.25 (0.05–1.33)
Cattle treatment	−0.88	0.52	0.091	0.41 (0.15–1.16)
Land type suitable for Arable use^e^	−0.82	0.62	0.182	0.44 (0.13–1.49)
Land type suitable for mixed agriculture^e^	0.62	0.53	0.241	1.86 (0.65–5.31)

a *Coeff, coefficient; SE, Standard error*.

b *(Log_10_) transformed*.

c *Square root transformed*.

d *PC1 (Principle Component 1) is an index variable created by Principal Components Analysis (Minitab v.18). PC1 explains 52.7% of the variation in the data. Larger values of PC1 represent higher number of holdings and density of cattle, sheep, and pigs*.

e *Land defined as suitable for arable use within James Hutton Land Capability for Agriculture (LCA) classification code (https://www.hutton.ac.uk/learning/exploringscotland/land-capability-agriculture-scotland)*.

The density of all cattle, sheep, and pigs, together with the density of their holdings across Scotland, were found to be significantly correlated (Supplementary Table 3) and therefore a Principle Component Analysis (Supplementary Figures 1, 2) was performed to compute independent variables for inclusion in the univariable model. Higher cattle and sheep density, higher cattle holding density, and the Principle Component 1 variable (PC1), which represented higher density and holding densities for all three species, were significantly associated with positive *intI1* herd status within the univariable model (Table 1, Supplementary Figure 3). Livestock density and livestock holding density were not significantly associated with *intI2* herd status.

We did not observe any significant association between the interpolated mean estimate for soil *intI1* abundance within the locality of a herd, derived from the National Soils Inventory of Scotland (NSIS2) soil *intI1* abundance dataset (49), and *intI1* status of that herd. However, by region, soil *intI1* abundance in the NSIS2 dataset was not uniformly distributed, with a substantial hotspot observed for high *intI1* abundance within the North East AHD (Supplementary Figures 4, 5). For *intI2*, only 3/183 soil locations yielded positive results and therefore comparison with the cattle herd data was not possible.

**Figure 2:**
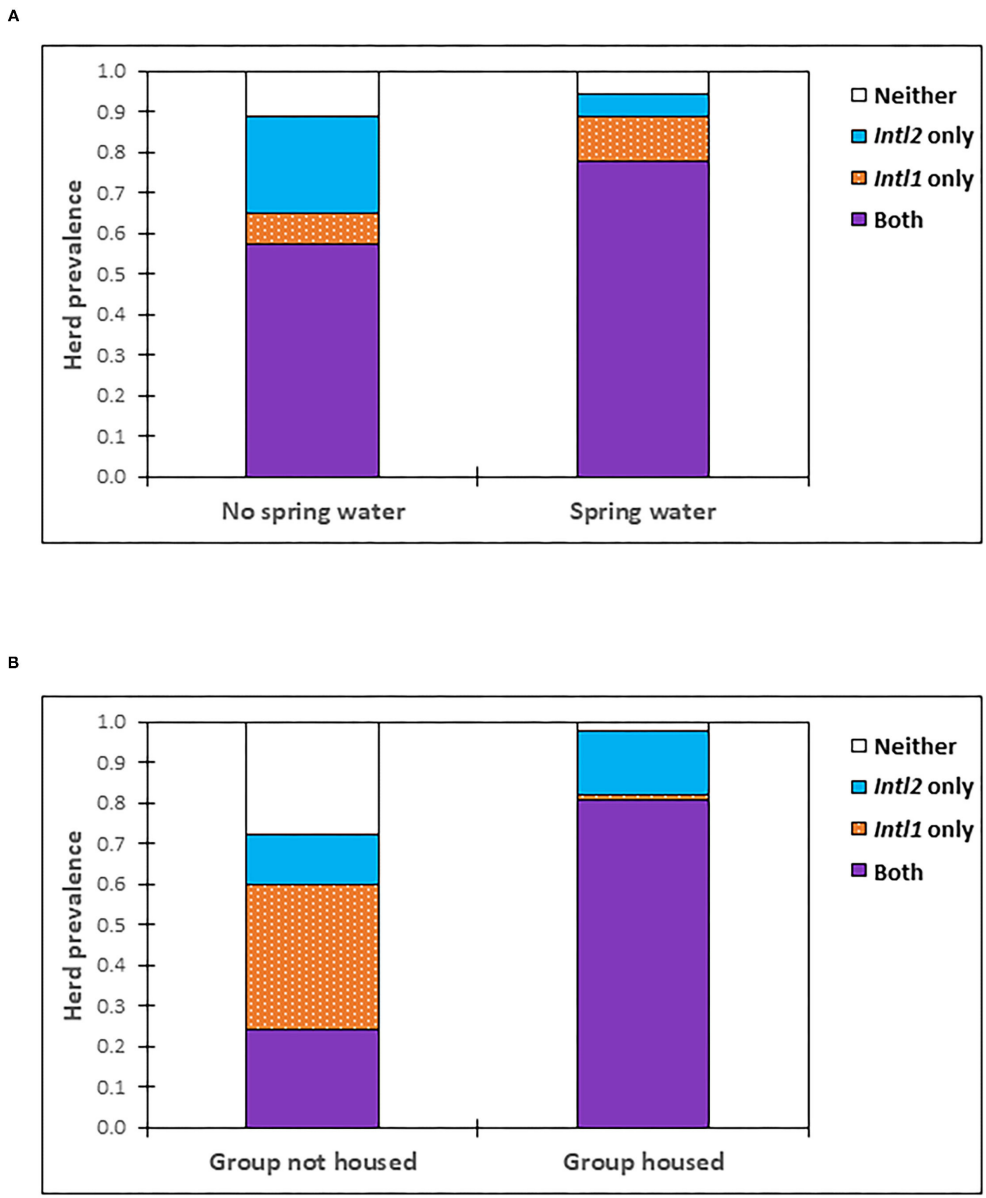
Prevalence of herds positive for *intI1* and/or *intI2* by **(A)** whether cattle are watered from a natural spring source, or not and **(B)** whether cattle are housed/not housed i.e., grazed, at the time of sampling.

After the backward selection process, the variables that were retained in the most parsimonious multivariable model for *intI1* included cattle watered from a natural spring source (*P* = 0.017; OR 4.40, 95% CI: 1.31–14.78) (Figure 2A), the North East and South East AHD, and Spring season of the year (Table 3). The only variable significantly associated with the detection of the *intI2* gene within the multivariable model was being housed at the time of sampling (*P* < 0.001; OR 75.01, 95% CI: 10.41–540.50) (Table 3, Figure 2B).

**Table 3:** Results of the multivariable generalized linear mixed model, showing region, season and herd management variables for herd *intI1* and *intI2* PCR-positive status, given as odds ratio with 95% confidence interval (CI).

**Response**	**Variable**	**Estimate**	**SE**	***P***	**Odds ratio (95% CI)**
***intI1***	**Animal Health District**				
	Central	1.24	0.88	0.165	3.45 (0.59–20.09)
	Islands	0.52	0.84	0.543	1.68 (0.30–9.06)
	North East	2.44	1.23	0.049	11.51 (1.01–130.89)
	South East	2.16	1.04	0.04	8.69 (1.11–20.88)
	South West	1.34	0.85	0.119	3.82 (0.70–20.88)
	Highland	–	–	–	–
	**Season**				
	Autumn	1.28	0.81	0.12	3.86 (0.71 – 18.09)
	Winter	0.11	0.74	0.883	1.12 (0.25 – 4.91)
	Spring	1.84	0.88	0.04	6.31 (1.10 – 36.37)
	Summer	–	–	–	–
	**Natural spring water source**				
	Yes	1.48	0.61	0.017	4.40 (1.31-14.78)
	No	–	–	–	–
***intI2***	**Animal Health District**				
	Central	0.46	1.58	0.773	1.58 (0.07 – 37.27)
	Islands	1.71	1.91	0.377	5.54 (0.11 – 269.40)
	North East	−0.96	1.32	0.467	0.38 (0.03 – 5.34)
	South East	0.38	1.43	0.793	1.46 (0.08 – 26.57)
	South West	1.03	1.34	0.449	2.79 (0.18 – 42.59)
	Highland	–	–	–	–
	**Season**				
	Autumn	1.42	1.07	0.193	4.12 (0.47 – 35.81)
	Winter	1.36	1.26	0.288	3.88 (0.31 – 48.85)
	Spring	1.66	1.47	0.263	5.25 (0.28 – 98.10)
	Summer	–	–	–	–
	**Group housed at sampling**				
	Yes	4.32	0.99	<0.001	75.01 (10.41 – 540.5)
	No	–	–	–	–

## Discussion

In this study we investigated the presence of the class 1 and 2 integron genes, *intI1* and *intI2*, in fecal samples from a national, cross-sectional survey of Scottish cattle herds. The prevalence of herds that tested positive by PCR for *intI1* and *intI2* genes was high, and co-occurrence of both genes within individual herds was common. These results provide a preliminary baseline for integron prevalence in Scottish cattle and for comparison with data from other farmed species, environmental or human settings. The ARG cassette structure of integrons enables dissemination of multiple antimicrobial resistance, therefore high prevalence of these elements has consequent implications for national policies to reduce antimicrobial resistance within agricultural settings. This is of particular relevance where ARG carriage may persist in the absence of direct selection pressures through linkage with other genes, such that restricting individual antimicrobial usage may not lead to a comparable reduction in resistance prevalence within livestock reservoirs.

To our knowledge, there are relatively few reports on the occurrence of integrase genes within agricultural settings in Scotland. For example, a previous longitudinal study identified *intI1*-positive commensal *E. coli* strains on a single organic cattle farm (53), as well as class 1 and 2 integrons in non-O157 *E.coli* from two conventional beef herds (54), however national cross-sectional data has so far been lacking. To date there have been relatively few culture-independent surveys to ascertain integron prevalence in livestock, although Barlow et al. (55) observed a similarly high PCR prevalence of class 1 and 2 integrons in cattle presenting at Australian abattoirs, and high prevalence has also recently been reported in cattle from France (44). Screening individual bacterial isolates from varied source populations and clinical collections is more common, and has highlighted the extensive distribution of class 1 integrons in diverse animal populations worldwide (56–58). Carriage of *intI1* and *intI2* by bovine intestinal bacteria therefore appears common, indicative of a substantial reservoir for these elements within cattle populations. We did not detect the class 3 integrase gene in any of the pool samples tested, which is consistent with cattle data from France (44). This was not unexpected: class 3 integrons have so far been reported in only a restricted range of bacterial species, within limited settings (9, 24, 25).

We observed evidence suggestive of a differential spatial distribution for *intI1*-positive herds across Scotland, with a significantly higher prevalence observed in the North East and South East, compared to the lowest prevalence region, the Highlands. Data on soil integron abundance (49) from the 2007–2010 survey of soil samples in Scotland also demonstrated a marked hotspot for *intI1* gene abundance in the North East region of Scotland (Supplementary Figure 5). This region also hosts the highest pig population and pig farm holding density in Scotland (59), as well as cattle holding density (60), and together with Central and South Eastern Scotland is an area with a higher proportion of land set to arable agricultural use. Within the univariable analysis, higher overall cattle, sheep and pig densities and livestock-holding densities, represented by PC1, were significantly associated with positive *intI1* herd status, suggesting that overall livestock density in an area may influence integron carriage. The inclusion of the interpolated and smoothed local soil *inti1* abundance estimates in the model did not highlight any direct association with positive herd integron status. However, the concurrence observed in the higher North East prevalence for *intI1* in the two differing sample datasets, cattle in this study and soil from the NSIS2 archive, albeit collected and assayed at different time periods using distinct methodologies, may not be due to chance alone and warrants further investigation. In contrast, no apparent spatial variation was observed in *intI2* herd status, with prevalence high in all regions. Compared to *intI1, intI2* was detected in only three locations in the NSIS2 dataset (Supplementary Figure 4), from regions where cattle prevalence was highest. This was not unexpected, since class 2 integrons are less commonly isolated from natural environments, with most reports relating to manure-enriched agricultural soils, or where land is contaminated with human-derived effluents (15).

Particular herd management factors were associated with an increased risk of both *intI1* and *intI2* positive status. For *intI1*, being housed at sampling, cattle movements into the herd, the number of cattle in a herd aged 12–30 months and a spring water source were all risk factors within the univariable model, although only cattle watered from a natural spring source was retained as a significant variable within the multivariable analysis. Cattle movement provides importation and mixing opportunities for bacteria and ARG within a herd; this variable was also found to be significantly associated with isolation of β-lactam and AmpC resistant *Enterobacteriacea* from a 20 herd subset within the Scottish BECS survey (61). These two complementary studies, the current culture-independent survey of 108 herds and the smaller, culture-based analysis by Velasova et al. (61), both suggest animal movements may correlate with ARG carriage within the BECS survey population. Lastly, cattle aged 12–30 months are typically finisher animals and the number of these animals in a herd may act simply by increasing the diversity and abundance of the local reservoir, providing a greater number of host compartment systems through which bacteria passage and multiply.

Watering cattle from natural spring water sources was retained as a significant factor for positive *intI1* herd status within the final multivariable model. Spring-sourced water in Scotland may be influenced by local geochemical factors such as rock composition, and subject to contamination through groundwater with environmental bacteria, pollutants and fertilizer. Cropland augmentation with manure or slurry increases the abundance of class 1 integrons in soils (62–65), as well as the overall level and diversity of ARGs (66). Whilst we did not observe any direct link between manure or slurry spreading and integron status on individual herd holdings in this study, our methodology was not able to capture local area information on waste-spreading practices. Effluent run-off from neighboring holdings, or wider dispersal of effluents into water-courses after heavy rainfall and through river flooding of land can influence the distribution and overall levels of agricultural pollutants, bacteria and ARGs in natural water supplies (67, 68). Integron abundance has also been shown to increase in river and spring water systems following rainfall events (69, 70) and has been detected at higher levels in agricultural watershed, than in the receiving waterbodies into which they discharge (71).

Housing was a strong predictor for positive *intI2* status; this effect was notably greater than observed with *intI1* in the univariable analysis and was maintained as a significant risk factor in the multivariable model. A “housed” effect has previously been reported for *intI2* in Australian beef cattle (72), in which higher *intI2* prevalence was observed by both PCR and culture-isolation in feces from grain-fed housed, compared to grazed animals. No difference in *intI1* prevalence was observed between housed and grazed cattle in the Australian data, with prevalence high in both groups. Given in our study we used a multiplex PCR system, we would anticipate that any direct influence from diet on fecal composition, such as the presence of differing fecal PCR inhibitors, would have also shown an equivalent effect on *intI1*. However, we observed no substantial difference in the minimum recorded C_t_ for *intI1* between housed and grazed herds, in marked contrast to the *intI2* response (see Supplementary Figure 6, Supplementary Table 8). Housing may influence bacterial colonization and ARG carriage through higher stocking density, with the proximity and build-up of manure and slurry providing more frequent opportunities for mixing and genetic exchange of mobile elements within bacterial populations (73). Agga et al. (74) demonstrated a spatial gradient decline in integron gene abundance based on linear distance from housing areas through to pasture-land in feedlot beef cattle, an effect that was maintained for 2 years following stock removal. Housed cattle typically receive grain-based feed, and it is possible that such diets may preferentially support colonization by bacteria hosting the *intI2* gene. Further, grain-based proprietary feeds are often formulated with additional trace metal elements such as copper, zinc, manganese, cobalt and selenium, or livestock may be supplemented with separate mineral formulations (75, 76). Copper, in particular, is often widely used within agricultural settings as fungicide, disinfectant and fertilizer. Evidence from recent studies suggests a possible role for agricultural copper in co-selection for antimicrobial resistance traits (77, 78), for example through linkage with copper-specific efflux pumps and the plasmid-borne copper resistance system (35). Bioinformatic analyses have also highlighted the close physical proximity and correlation between integrons and metal resistance genes in bacterial genomes (31, 79), as seen with the metal efflux pump gene *czcA* (7, 80) and the *mer* genes conferring resistance to mercury (81). The possibility that integron carriage may be influenced by exposure to heavy metals through diet or topical administration, as well as biocide use when stock are housed, merits further exploration.

We used pooled fecal DNA extracts, since testing every individual pat sample in a herd was beyond the scope of this study. Pooling is recognized as an efficient and cost-effective method when screening cattle herds for pathogens (41, 42, 82). Where identification of carriage by individual animals or samples is not required in a first screen, pooling can maintain sensitivity at the herd level, dependant on the expected prevalence (42, 83). In this study we identified the majority of herds positive for *intI1* and *intI2* within the first two pools screened per herd, and therefore suggest herd pool screening as a useful tool that could be employed for large scale surveillance of common ARGs within livestock populations.

In conclusion, we have demonstrated widespread and high prevalence of the integrase *intI1* and *intI2* genes in Scottish cattle herds. This data suggest that integrons are ubiquitous within cattle populations across Scotland. Statistically significant associations with positive herd status were observed, including the North East region and natural spring water source for *intI1*, and housed status for *intI2*. These associations are plausible, within the context of existing knowledge. The results of this study may direct future, more detailed investigations into carriage of integrons and other individual resistance genes, with hypotheses based upon those variables found to be of significance, such as location, housing status, and water source. The role of integrons as vehicles for facilitating horizontal transmission and retention of ARGs within livestock reservoirs, together with factors that may influence their carriage, require further exploration.

## Data Availability Statement

The original contributions presented in the study are included in the article/Supplementary Material, further inquiries can be directed to the corresponding author.

## Author Contributions

CFR and DH: conceptualization, design and funding acquisition, and wrote the original draft manuscript. DH: supervision and project administration. CFR, DH, OB, NS, and CC: contributed resources. CFR, DH, and OB: methodology. CFR and HW: performed the laboratory analyses. CFR, HW, DH, MC-T, ST, TP, DE, and CK: data acquisition and/or curation. TP and MC-T: performed the formal statistical analyses and visualization. CFR, TP, MC-T, CK, HW, OB, ST, NS, CC, DE, and DH: writing—review and editing. All authors contributed to read and approved the submitted version.

## Funding

CFR was funded by an Erasmus +: Erasmus Mundus Scholarship from the European Union [EU Grant Agreement: 2016–2071], with additional salary support to CFR and HW provided through an Institute Strategic Programme grant from the Biotechnology & Biological Sciences Research Council [BBS/E/D/20002173]. DH was funded through a personal fellowship from the Wellcome Trust [105832/Z/14/Z]. TP thanks the French National Research Agency and Boehringer Ingelheim Animal Health France for support through the IDEXLYON project (ANR-16-IDEX-0005) and the Industrial Chair in Veterinary Public Health, Lyon, France. The collection of the original cattle samples used in this study was funded by Food Standards Scotland and the Food Standards Agency [Project number FS101055: *E. coli* O157 super-shedding in cattle and the mitigation of human risk]. This research was funded in part by the Wellcome Trust [105832/Z/14/Z].

## Conflict of Interest

The authors declare that the research was conducted in the absence of any commercial or financial relationships that could be construed as a potential conflict of interest.

## Publisher's Note

All claims expressed in this article are solely those of the authors and do not necessarily represent those of their affiliated organizations, or those of the publisher, the editors and the reviewers. Any product that may be evaluated in this article, or claim that may be made by its manufacturer, is not guaranteed or endorsed by the publisher.
