# Supplementary material for: High Prevalence and Factors Associated With the Distribution of the Integron intI1 and intI2 Genes in Scottish Cattle Herds
Source: Front Vet Sci. 2021 Oct 29;8:755833. doi: 10.3389/fvets.2021.755833 (PMC8585936; doi:10.3389/fvets.2021.755833)
Supplement: Supplementary file 1 [file Data_Sheet_1.docx]

**Table S1.** Primer and probe sequences for the *intI1, intI2* and *intI3* multiplex real-time PCR assay, from Barraud *et al.* (2010).

| **Target** | **Primer and probe sequences, 5’ to 3’** | | **Product size (bp)** |
| --- | --- | --- | --- |
| ***intI1*** | **Forward** | GCCTTGATGTTACCCGAGAG | 196 |
|  | **Reverse** | GATCGGTCGAATGCGTGT |  |
|  | **Probe** | 5’-FAM-ATTCCTGGCCGTGGTTCTGGGTTTT-BHQ1 |  |
| ***intI2*** | **Forward** | TGCTTTTCCCACCCTTACC | 195 |
|  | **Reverse** | GACGGCTACCCTCTGTTATCT C |  |
|  | **Probe** | Texas Red-TGGATACTCGCAACCAAGTTATTTTTAGCTG- BHQ2 |  |
| ***intI3*** | **Forward** | GCCACCACTTGTTTGAGGA | 138 |
|  | **Reverse** | GGATGTCTGTGCCTGCTTG |  |
|  | **Probe** | CY5-CGCCACTCATTCGCCACCCA-BHQ3 |  |

**Reference:**

Barraud O, Baclet MC, Denis F, Ploy MC. Quantitative multiplex real-time PCR for detecting class 1, 2 and 3 integrons. J Antimicrob Chemother (2010) 65(8):1642-5. doi: 10.1093/jac/dkq167. Epub 2010 Jun 11. PMID: 20542899.

**Pool Samples and Technical Replicates**

A total of 386 pools of faecal DNA extracts, representing 1829 faecal pat samples collected from 108 herds, were tested by real-time PCR for the *intI1*, *intI2* and *intI3* genes*.* The median number of pools tested per herd was 2.5 (range 2 – 13), which represented a median of 10.5 faecal pat samples tested per herd (range 7 – 61). The proportion of positive pools was 41.7% (161/386) for *intI1* and 54.4% (210/386) for *intI2*; 32.4% of pools (125/386) were positive for both *intI1* and *IntI2*, and 36.3% (140/386) of pools were negative for both genes. No pools were positive for the *intI3* gene.

Technical replicates (TR) in which a repeat PCR was performed using the same DNA pool sample in a different assay run, were performed for 219 pool samples. Pool sample status between replicates concurred as both negative/ both positive for *intI1* in 87.7% of pools and *intI2* in 94.1% pools. Discordant pools did not affect herd status where another pool in the herd was already positive, with overall 105/108 herds (97.2%) agreeing herd status between all replicates for both *intI1* and *intI2*. For the final allocation of herd status, the mean TR C_t_ value between runs was calculated for each pool sample that was repeated and used to allocate pool as positive or negative, based on the PCR cut-off, as described in the Methods.

**Table S2.** The mean of TR mean C_t_ values by positive/negative status and *intI* class, with the mean and standard deviation (SD) for within replicate C_t_ SD for all replicates returning a C_t_ value.

|  | **n,**  **pools** | **Mean of**  **TR C_t_ mean** | **Mean of**  **TR C_t_ SD** | **SD of**  **TR C_t_ SD** |
| --- | --- | --- | --- | --- |
| ***intI1*** | 219 |  |  |  |
| Positive | 95 | 29.29 | 0.53 | 0.41 |
| Negative, with C_t_^1^ | 114 | 34.35 | 0.92 | 1.03 |
| ***intI2*** | 219 |  |  |  |
| Positive | 133 | 30.13 | 0.52 | 0.37 |
| Negative, with C_t_^2^ | 71 | 36.38 | 0.94 | 0.81 |

^1^Additional 10 negative pool samples returned no C_t_ value, all negative samples had a previously confirmed positive result by PCR to Phocine herpes virus glycoprotein B or the Shiga toxin protein *stx* gene (Hoyle *et al.* 2021)

^2^Additional 15 negative pool samples returned no C_t_ value, all negative samples had a previously confirmed positive result by PCR to Phocine herpes virus glycoprotein B or the Shiga toxin protein *stx* gene (Hoyle *et al.* 2021)

**Reference:**

Hoyle, D.V.; Keith, M.; Williamson, H.; Macleod, K.; Mathie, H.; Handel, I.; Currie, C.; Holmes, A.; Allison, L.; McLean, R.; Callaby, R.; Porphyre, T.; Tongue, S.C.; Henry, M.K.; Evans, J.; Gunn, G.J.; Gally, D.L.; Silva, N.; Chase-Topping, M.E. Prevalence and Epidemiology of Non-O157 Escherichia coli Serogroups O26, O103, O111, and O145 and Shiga Toxin Gene Carriage in Scottish Cattle, 2014-2015. Appl Environ Microbiol. 2021 Apr 27;87(10):e03142-20. doi: 10.1128/AEM.03142-20. PMID: 33712425; PMCID: PMC8117755.

**Principal Components Analysis: Animal Density Variables**

Within the study we examined the density of cattle, pigs and sheep, as well as the density of their holdings, in the region of each herd that was sampled in this study (see Methods section for details). Pearson correlation across the six variables showed significant correlation among most of the variables (Table S3). As a result, a Principal Component Analysis (PCA) was performed in Minitab version 18 to create independent variables to use in the risk factor analysis. PCA was run using a correlation matrix after transforming the variables. There were two factors (PC1 and PC2) with eigenvalues greater than 1 that explained 72.1% of the variation in the data. PC1 explained 57.1% of the variation and represented increasing density of animals and holdings. PC2 explained 19.4% of the variation in the data and represented cattle and sheep (positive values) versus pig (negative values) (Figure S1). PC1 and PC2 were included in the risk factors analysis (see main manuscript). Figure S2 shows a plot of PC1 and PC2 overlaid by the *intI* status of the herd (i.e. both *intI1* and *intI2* positive; neither *intI1* or *intI2* positive; *intI1* only positive, *intI2* only positive). Only PC1 was carried forward into the multivariable model. Herds that were *intI2* positive had lower values of PC1 compared to herds which were *intI1* positive, which had higher values of PC1 (Figure S3).

**Table S3**. Pearson correlation of the six animal density variables examined in this study, lower left quadrant. Top value is the Pearson correlation value and the lower value is the *P*-value. HDensity: holding density.

|  | **Cattle Density** | **Cattle HDensity** | **Pig Density** | **Pig HDensity** | **Sheep Density** | **Sheep HDensity** |
| --- | --- | --- | --- | --- | --- | --- |
| **Cattle Density** | 1.00 |  |  |  |  |  |
| **Cattle HDensity** | 0.806  <0.001 | 1.00 |  |  |  |  |
| **Pig Density** | 0.077  0.429 | 0.260  0.007 | 1.00 |  |  |  |
| **Pig HDensity** | 0.454  <0.001 | 0.718  <0.001 | 0.599  <0.001 | 1.00 |  |  |
| **Sheep Density** | 0.350  <0.001 | 0.293  <0.001 | 0.068  0.582 | 0.343  <0.001 | 1.00 |  |
| **Sheep HDensity** | 0.313  0.001 | 0.622  <0.001 | 0.154  0.112 | 0.596  <0.001 | 0.556  <0.001 | 1.00 |

**Figure S1.** Loading plot of principal component results on six animal density variables. PC1 explains 57.1% of the variation in the data and represents higher density of all animals and holdings. PC2 explains 19.4% of the variation in the data, separating cattle and sheep (positive values) from pigs (negative values).


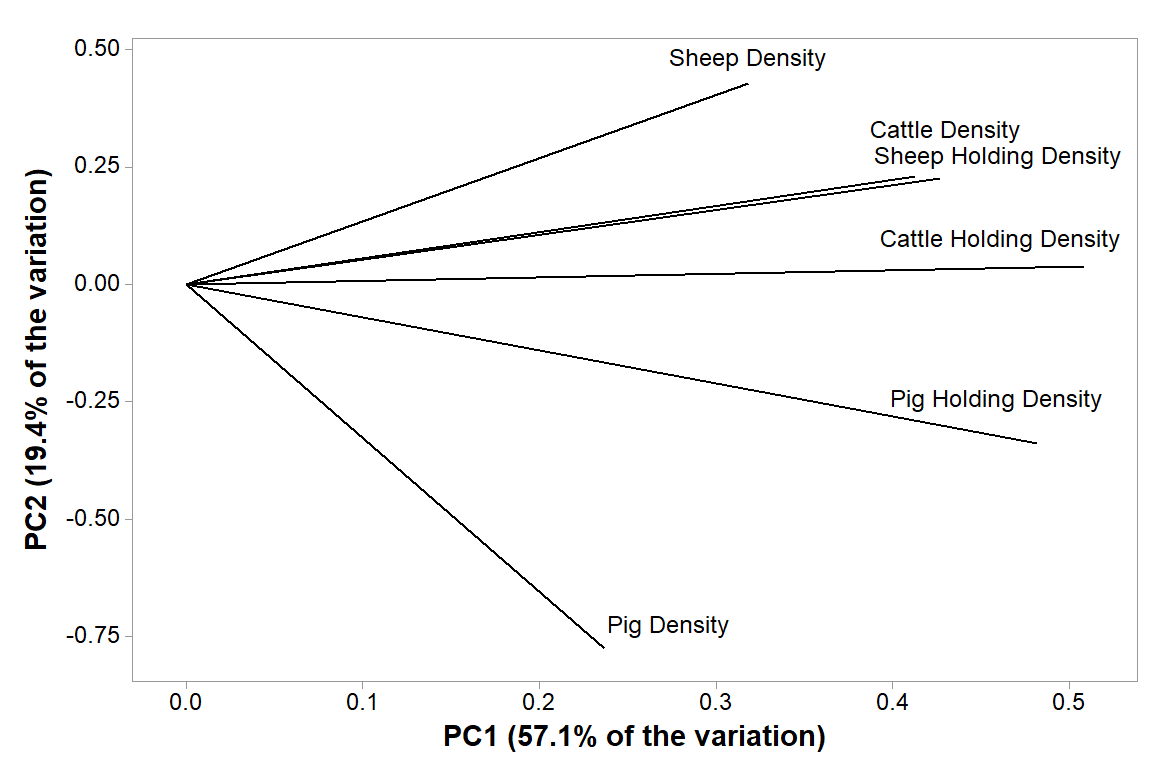


**Figure S2.** Distribution of data for PC1 and PC2 from Principal Components Analysis with ellipses corresponding to the herd *intI* status (i.e. Both *intI1* and *intI2* positive; Neither *intI1* or *intI2* positive; *intI1* positive only and *intI2* positive only). Ellipses are 68% prediction ellipse (alpha=0.32) because 68% is the probability of observing univariate normal data that is within one standard deviation of the mean.


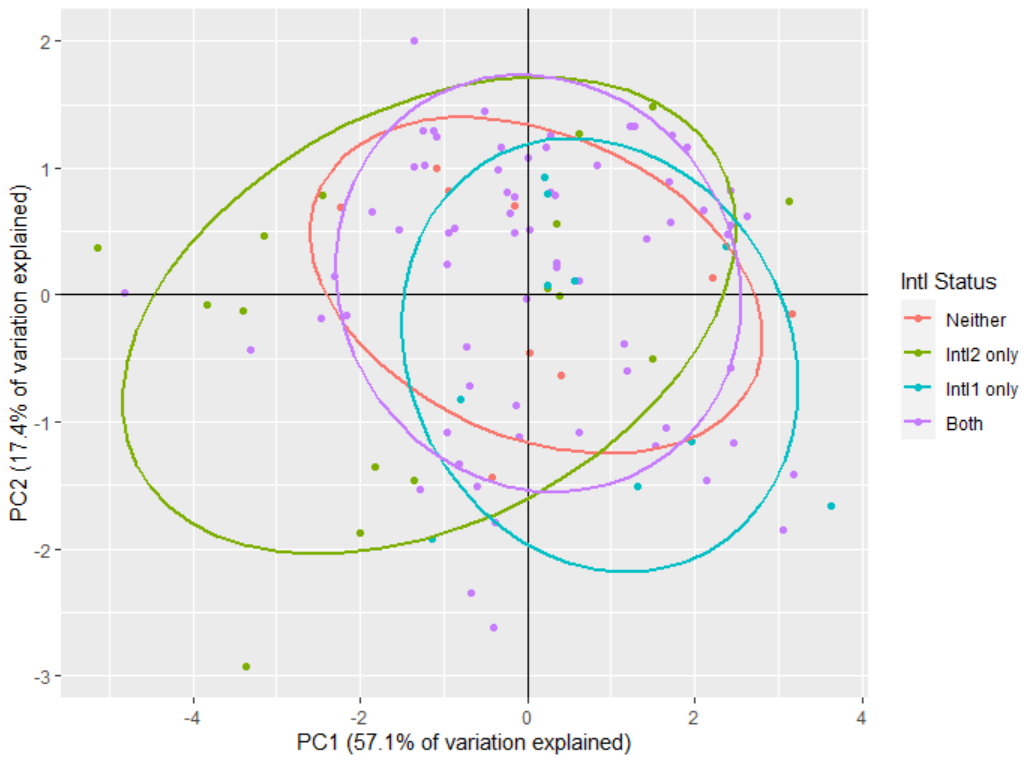


**Figure S3.** Boxplot of PC1 values for herds that were Both *intI1* and *intI2* positive; Neither *intI1* or *intI2* positive; *intI1* positive only and *intI2* positive only. Black circles represent individual data points.


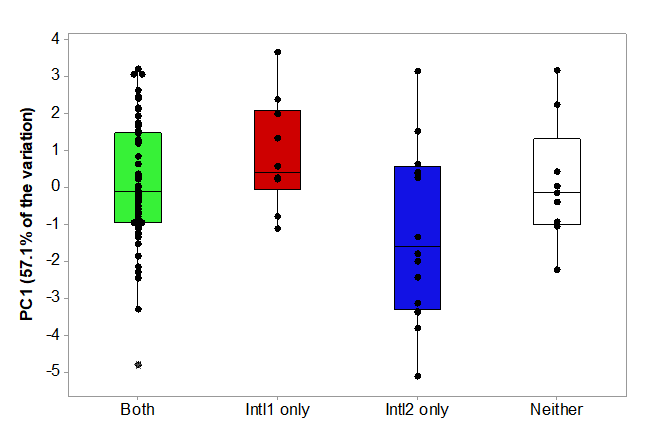


**Figure S4.** Spatial distribution of integron *intI1* and *intI2* genes in soil samples from Scotland collected over the period 2007-2010, shown as relative gene abundance per total bacteria, from the dataset of Knapp *et al.* (2019). Animal Health District boundaries are marked by solid lines.


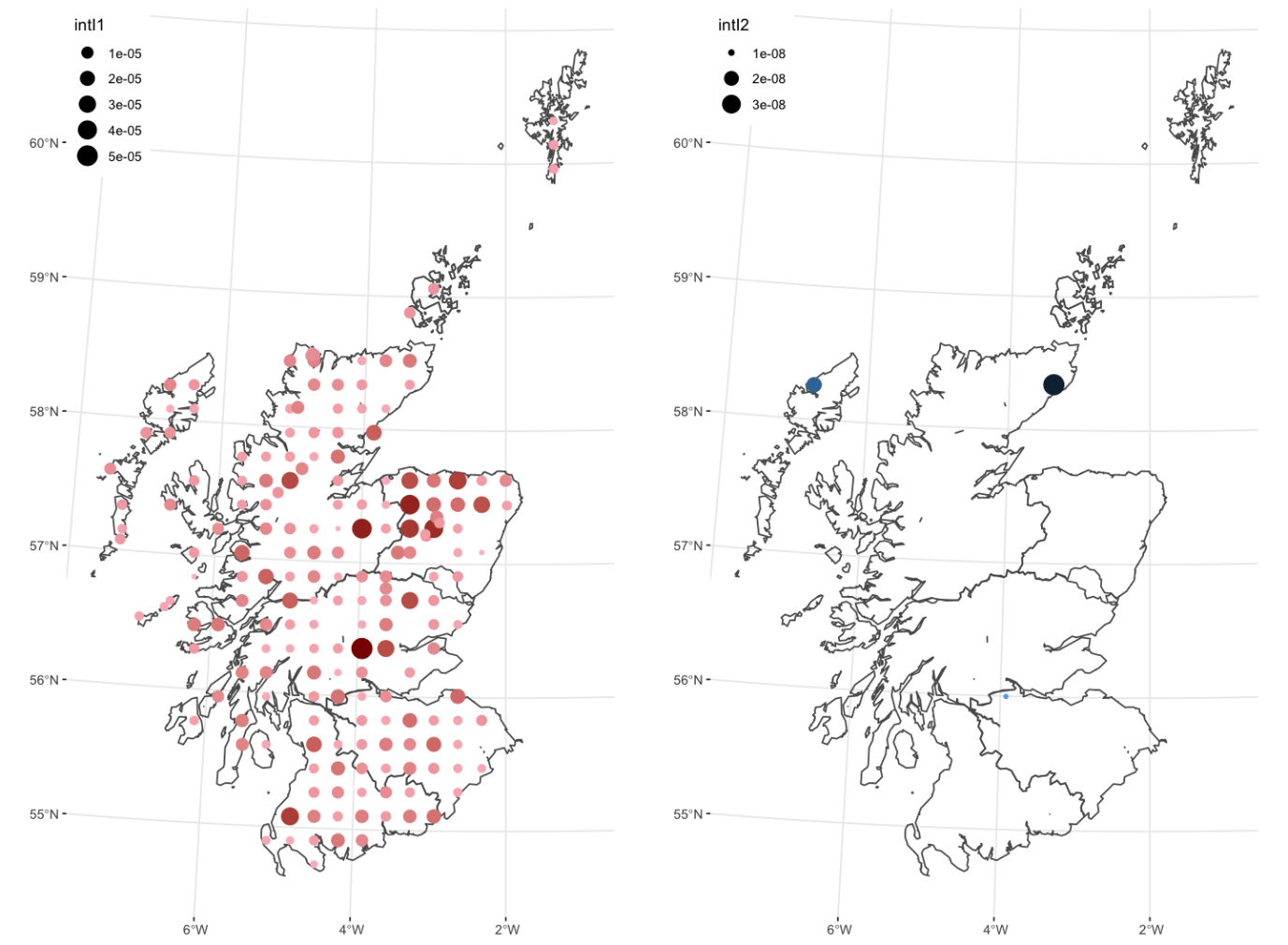


**Reference:**

Knapp CW, Graham DW, Freitag T, Pagaling E, Hough R, Avery L, Zhu Y-G, Su J, Zhou XY. Antibiotic resistance genes found in soils across the entire Scottish landscape (2007-10). NERC Environmental Information Data Centre. (2019) https://doi.org/10.5285/d3498e93-4ac5-4eab-bc1a-eb2328771d24

**Figure S5.** Kernel smoothed distribution of integron *intI1* gene in soil samples from Scotland collected over the period 2007-2010, shown as relative gene abundance per total bacteria and smoothed using a bandwidth parameter of 50 km, from the dataset of Knapp *et al.* (2019). Animal Health District boundaries are marked by solid lines.


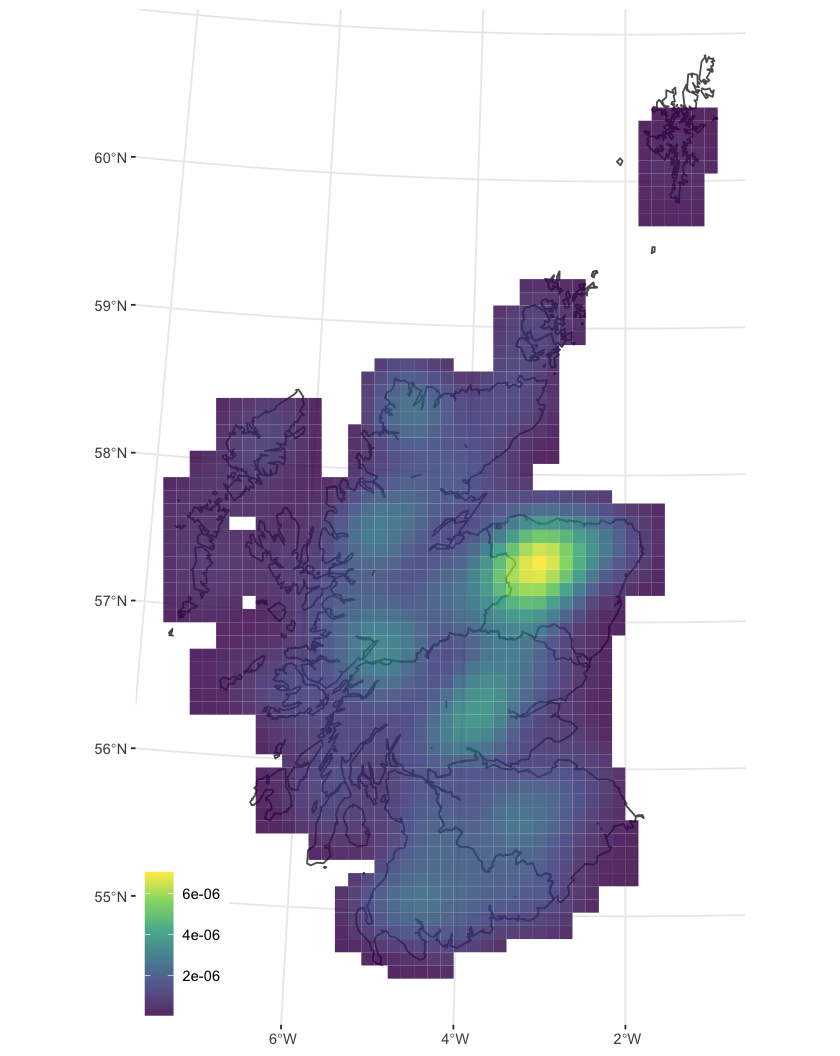


**Reference:**

Knapp CW, Graham DW, Freitag T, Pagaling E, Hough R, Avery L, Zhu Y-G, Su J, Zhou XY. Antibiotic resistance genes found in soils across the entire Scottish landscape (2007-10). NERC Environmental Information Data Centre. (2019) https://doi.org/10.5285/d3498e93-4ac5-4eab-bc1a-eb2328771d24

**Table S4.** List of herd geographic, demographic and management factors included as variables in the risk factor analysis, showing the number of herds (n) present in each category (for categorical variables) out of a total of 108 herds, or the median value and interquartile range (IQR) for continuous variable measurements.

| **Continuous Variables** | **Median (IQR)** |
| --- | --- |
| Number of cattle age less than 1 year | 50 (80) |
| Number of cattle 12 to 30 months | 32 (63) |
| Total number of cattle | 170 (221) |
| Number of heifers | 8 (20) |
| Number of cows | 49 (80) |
| Land type: LCA code^1^ | 3.2 (1.0) |
| Soil *intI1* level^2^ | 1 x10^-6^(1x10^-6^) |
| Pig density | 0.67 (10.7) |
| Density of pig holdings | 6.6 (10.7) |
| Cattle density | 42.8 (30.3) |
| Density of cattle holdings | 28.0 (19.1) |
| Sheep density | 102.2 (95.3) |
| Density of sheep holdings | 23.3(15.0) |
| Principal Component 1 (PC1)^3^ | -0.014 (2.51) |
| Principal Component 2 (PC2)^3^ | 0.246 (1.67) |
| **Categorical Variables** | **Number of herds** |
| **Animal Health District**  Central  Highlands  Islands  North East  South East  South West | 16  18  16  19  17  22 |
| **Season**  Autumn  Winter  Spring  Summer | 33  24  28  23 |
| **Herd type**  Dairy  Suckler beef  Special finisher  Other, not as above | 13  71  14  10 |
| **Other animals present on farm**  Sheep  Wildfowl  Poultry  Wild geese  Gulls  Breeding bulls  Foster calves | 71  11  29  48  36  31  12 |
| **Water supply (may have more than one source)**  Cattle: mains  Cattle: spring  Cattle: natural  Cattle: natural crosses another farm  Farmhouse: mains  Farmhouse: private | 69  54  72  47  78  32 |
| **Cattle health**  Cattle health problems  Cattle received treatment | 7  38 |
| **Management - Other**  Brought on other cattle  Always isolate brought on  Brought on livestock other than cattle  Livestock not owned on farm  Manure spreading  Slurry spreading  Cattle housed at sampling  Cattle housed with cattle < 1 year old  Certified Organic  Change of feed  Change of location  No calving in herd  Calve all year | 85  57  58  19  66  46  83  37  5  25  19  14  16 |
| **Land Type^1^**  Arable land  Mixed Agriculture  Improved grassland  Rough grazing land | 17  75  11  5 |
| **Soil Type^4^**  Soil Type  Alluvial  Brown  Peat  Mineral  Mineral gleys  Mineral podzols  Humus-iron podzols (HIP) | (see “Land type: LCA code”)  4  44  6  54  31  23  29 |

**^1^**According to the Macaulay Land Capability for Agriculture classification code, James Hutton Institute, Scotland, UK (<https://www.hutton.ac.uk/learning/exploringscotland/land-capability-agriculture-scotland>)

**^2^** From Knapp CW, Graham DW, Freitag T, Pagaling E, Hough R, Avery L, Zhu Y-G, Su J, Zhou XY. Antibiotic resistance genes found in soils across the entire Scottish landscape (2007-10). NERC Environmental Information Data Centre. (2019) https://doi.org/10.5285/d3498e93-4ac5-4eab-bc1a-eb2328771d24

**^3^**Principle component factors were calculated to quantify the six correlated cattle, sheep and pig density and holding density variables, see Table S7, Figures S4-6.

**^4^**General soil type defined by James Hutton Institute, Scotland, UK (<https://www.hutton.ac.uk/learning/soilshutton/soil-classification>)

**Table S5.** Overall, regional and seasonal herd-level prevalence estimates (95% confidence intervals, CI) for the *intI1* and *intI2* genes by real-time PCR, Scotland 2014 - 2015, within a generalized linear model (GLM); n, number of herds positive for *intI1* and *intI2* genes.

| **Variable** | ***intl1*** | | ***intl2*** | |
| --- | --- | --- | --- | --- |
|  | **n** | **Prevalence (CI)** | **n** | **Prevalence (CI)** |
| **Overall** | 83 | 76.85 (67.82 – 83.95) | 89 | 82.41 (73.90 – 88.57) |
| **Animal Health District** |  |  |  |  |
| Central | 12 | 75.0 (47.94 – 90.72) | 12 | 75.0 (47.61 – 90.83) |
| Highlands | 10 | 55.56 (31.75 – 77.05) | 15 | 83.33 (57.98 – 94.77) |
| Islands | 11 | 68.75 (41.97 – 87.00) | 15 | 93.75 (65.40 – 99.17) |
| North East | 18 | 94.74 (69.77 – 99.29) | 15 | 78.95 (54.16 – 92.25) |
| South East | 15 | 88.24 (62.19 – 97.16) | 14 | 82.35 (56.06 – 94.47) |
| South West | 17 | 77.27 (54.65 – 90.56) | 18 | 81.82 (59.30 – 93.29) |
| **Season** |  |  |  |  |
| Autumn | 28 | 84.85 (67.84 – 93.70) | 25 | 75.76 (57.73 – 87.73) |
| Winter | 16 | 66.67 (45.21 – 82.90) | 22 | 91.67 (71.51 – 97.97) |
| Spring | 24 | 85.71 (66.96 – 94.67) | 26 | 92.86 (75.0 – 98.26) |
| Summer | 15 | 65.22 (43.34 – 82.13) | 16 | 69.57 (47.38 – 85.30) |

**Table S6.** Univariable analysis of continuous variables for the *intI1* (A) and *intI2* (B) herd-level status by real-time PCR, Scotland 2014 - 2015, within a generalized linear model (GLM), for variables including Animal Health District region, season, herd management factors and environmental variables. Variables where *P* < 0.25 are italicized and highlighted in bold typeface. Displaying the median value and interquartile range (IQR) for continuous variable measurements; where “+” indicates herd positive, “-“ indicates herd negative; total number of herds sampled, n=108.

1. ***intI1***

| **VARIABLE** | **Median (IQR*)** | | **DF*** | **F*** | ***P**** | **BIC*** |
| --- | --- | --- | --- | --- | --- | --- |
|  | ***intI1*+ (n=83)** | ***intI1*- (n=25)** |  |  |  |  |
| Number of cattle age less than 1 year^1^ | 53 (83) | 50 (81) | 1,106 | 0.77 | 0.382 | 125.48 |
| ***Number of cattle 12 to 30 months***^1^ | 44 (71) | 11 (24) | 1,106 | 6.47 | ***0.012*** | 119.49 |
| ***Total number of cattle***^1^ | 180(224) | 122 (157) | 1,106 | 3.61 | ***0.060*** | 122.55 |
| Number of heifers^1^ | 7 (20) | 10 (16) | 1,106 | 0.24 | 0.622 | 125.99 |
| Number of cows^1^ | 50 (80) | 48 (66) | 1,106 | 0.00 | 0.984 | 126.23 |
| Land type: LCA code^2^ | 3.2 (1.0) | 4.1 (1.0) | 1,106 | 0.04 | 0.839 | 126.19 |
| Soil *intI1* level^1^ | 1x10^-6^ (1x10^-6^) | 2x10^-6^ (1x10^-6^) | 1,106 | 0.00 | 0.970 | 126.23 |
| ***Pig density***^1^ | 0.70 (13.6) | 0.57 (8.2) | 1,106 | 1.79 | ***0.184*** | 124.35 |
| Density of pig holdings^3^ | 6.65 (6.34) | 6.14 (8.2) | 1,106 | 0.70 | 0.405 | 125.52 |
| ***Cattle density***^3^ | 47.1 (30.7) | 29.4 (36.7) | 1,105 | 6.40 | ***0.013*** | 118.47 |
| ***Density of cattle holdings***^3^ | 30.2 (17.4) | 23.7 (15.8) | 1,105 | 4.34 | ***0.040*** | 121.07 |
| ***Sheep density***^1^ | 105 (93) | 70 (105) | 1,106 | 4.43 | ***0.038*** | 121.59 |
| Density of sheep holdings^3^ | 23.0 (13.1) | 23.6 (21.7) | 1,106 | 0.58 | 0.450 | 125.66 |
| ***Principal Component 1 (PC1)***^4^ | 0.012 (2.45) | -0.422 (2.86) | 1,105 | 4.99 | ***0.028*** | 120.37 |
| Principal Component 2 (PC2)^4^ | 0.319 (1.96) | 0.052 (1.29) | 1,105 | 0.16 | 0.690 | 125.52 |

1. ***intI2***

| **VARIABLE** | **Median (IQR*)** | | **DF*** | **F*** | ***P**** | **BIC*** |
| --- | --- | --- | --- | --- | --- | --- |
|  | ***intI2*+ (n=89)** | ***intI2*- (n=19)** |  |  |  |  |
| Number of cattle age less than 1 year^1^ | 50 (78) | 53 (88) | 1,106 | 0.05 | 0.892 | 109.79 |
| Number of cattle 12 to 30 months^1^ | 32 (66) | 19 (50) | 1,106 | 0.03 | 0.857 | 109.81 |
| Total number of cattle^1^ | 178 (207) | 138 (259) | 1,106 | 0.44 | 0.508 | 109.40 |
| Number of heifers^1^ | 8 (20) | 8 (20) | 1,106 | 0.16 | 0.694 | 109.68 |
| Number of cows^1^ | 50 (79) | 35 (90) | 1,106 | 0.57 | 0.452 | 109.28 |
| Land type: LCA code^2^ | 3.2 (1.0) | 3.2 (1.1) | 1,106 | 0.34 | 0.561 | 109.49 |
| Soil *intI1* level^1^ | 1x10^-6^ (1x10^-6^) | 2x10^-6^ (1x10^-6^) | 1,106 | 0.88 | 0.349 | 108.93 |
| ***Pig density^1^*** | 0.54 (7.84) | 2.86 (14.6) | 1,106 | 1.59 | ***0.210*** | 108.25 |
| ***Density of pig holdings^3^*** | 6.14 (5.99) | 9.11 (9.93) | 1,106 | 3.18 | ***0.078*** | 106.58 |
| Cattle density^3^ | 42.8 (31.2) | 45.0 (33.9) | 1,105 | 0.65 | 0.422 | 108.78 |
| Density of cattle holdings^3^ | 28.9 (19.6) | 25.4 (25.9) | 1,105 | 1.29 | 0.259 | 108.11 |
| Sheep density^1^ | 102.3 (97.9) | 95.5 (119.5) | 1,106 | 0.11 | 0.737 | 109.72 |
| Density of sheep holdings^3^ | 22.9 (16.6) | 24.8 (11.9) | 1,106 | 0.64 | 0.425 | 109.19 |
| ***Principal Component 1 (PC1)^4^*** | -0.148 (2.60) | 0.239 (2.77) | 1,105 | 1.83 | ***0.179*** | 107.51 |
| Principal Component 2 (PC2)^4^ | 0.404 (1.70) | 0.079 (1.86) | 1,105 | 0.91 | 0.342 | 108.53 |

*IQR, Interquartile range; DF, degrees of freedom (numerator DF, denominator DF); F, F-statistic; P, *P*-value; BIC, Bayesian Information Criterion.

^1^(Log_10_) transformed

^2^According to the Macaulay Land Capability for Agriculture classification code, James Hutton Institute, Scotland, UK (https://www.hutton.ac.uk/learning/exploringscotland/land-capability-agriculture-scotland)

^3^Square root transformed

^4^Principle component factors were calculated to quantify the six correlated cattle, sheep and pig density and holding density variables, see Table S7, Figures S4-6.

**Table S7.** Univariable analysis of categorical variables for the *intI1* (A) and *intI2* (B) herd-level status by real-time PCR, Scotland 2014 - 2015, within a generalized linear model (GLM), for variables including Animal Health District region, season, herd management factors and environmental variables. Variables where *P* < 0.25 are italicized and highlighted in bold typeface. Displaying number of herds within each category, where “Y” indicates “Yes” category, “N” indicates “No” category, “+” herd positive, “-“ herd negative; total number of herds sampled, n=108.

1. ***intI1***

| **VARIABLE** | **n*** | | **DF*** | **F*** | ***P**** | **BIC*** |
| --- | --- | --- | --- | --- | --- | --- |
|  | ***intI1*+ (n=83)** | ***intI1*-**  **(n=25)** |  |  |  |  |
| ***Animal Health District***  (Central) 1  (Highlands) 2  (Islands) 3  (North East) 4  (South East) 5  (South West) 6 | 12  10  11  18  15  17 | 4  8  5  1  2  5 | 5,102 | 1.65 | ***0.154*** | 134.43 |
| ***Season***  (Autumn) 1  (Winter) 2  (Spring) 3  (Summer) 4 | 28  16  24  15 | 5  8  4  8 | 3,104 | 1.77 | ***0.157*** | 130.04 |
| Herd type  Dairy Y  N  Suckler beef Y  N  Special finisher Y  N | 10  73  53  30  11  72 | 13  22  18  7  3  22 | 1,106  1,106  1,106 | 0.00  0.56  0.03 | 0.995  0.455  0.871 | 126.23  125.65  126.21 |
| Other animals on farm  Sheep Y  N  Wildfowl Y  N  ***Poultry*** Y  N  ***Wild geese*** Y  N  Gulls Y  N  Breeding bulls Y  N  Foster calves Y  N | 56  27  10  73  25  58  32  51  27  56  26  57  10  73 | 15  10  1  24  4  21  16  19  9  16  5  20  2  23 | 1,106  1,106  1,106  1,106  1,106  1,106  1,106 | 0.47  1.23  2.45  4.83  0.10  1.18  0.31 | 0.493  0.271  ***0.121***  ***0.030***  0.748  0.280  0.576 | 125.76  124.61  123.44  *121.19*  126.13  124.96  125.89 |
| Water supply  Cattle: mains Y  N  ***Cattle: spring*** Y  N  Cattle: natural Y  N  ***Cattle: natural crosses another farm*** Y  N  Farmhouse: mains Y  N  ***Farmhouse: private*** Y  N | 51  32  48  35  57  26  39  44  58  25  27  56 | 18  7  6  19  15  10  8  17  20  5  5  20 | 1,106  1,106  1,106  1,106  1,106  1,106 | 0.92  8.03  0.65  1.73  0.97  1.41 | 0.340  ***0.006***  0.423  ***0.192***  0.328  ***0.237*** | 125.28  117.09  125.60  124.44  125.20  124.70 |
| Cattle health  Cattle health Y  N  Cattle treatment Y  N | 6  77  29  54 | 1  24  9  16 | 1,106  1,106 | 0.32  0.01 | 0.572  0.923 | 125.87  126.22 |
| Management - Other  ***Brought on other cattle*** Y  N  Always isolate Y  N  Brought on livestock other than cattle Y  N  Livestock not owned on farm Y  N  Manure Y  N  ***Slurry*** Y  N  ***Cattle housed at sampling*** Y  N  Cattle housed with cattle < 1 year old Y  N  Certified Organic Y  N  ***Change feed*** Y  N  ***Change location*** Y  N  No calving Y  N  Calve all year Y  N | 70  13  44  39  47  36  14  69  50  33  38  45  68  15  29  54  4  79  22  61  17  66  12  71  12  71 | 15  10  13  12  11  14  5  20  16  9  8  17  15  10  8  17  1  24  3  22  2  23  2  23  4  21 | 1,106  1,106  1,106  1,106  1,106  1,106  1,106  1,106  1,106  1,106  1,106  1,106  1,106 | 6.33  0.01  1.22  0.13  0.11  1.47  4.93  0.07  0.03  2.15  1.91  0.69  0.04 | ***0.013***  0.929  0.272  0.719  0.736  ***0.228***  ***0.029***  0.787  0.865  ***0.146***  ***0.170***  0.408  0.850 | 120.08  126.23  125.00  126.11  126.12  124.71  121.45  126.16  126.20  123.71  126.86  125.45  126.20 |
| **Land Type**^1^  Arable land Y  N  Mixed Agriculture Y  N  Improved grassland Y  N  Rough grazing land Y  N | 13  70  57  26  9  74  4  79 | 4  21  18  7  2  23  1  24 | 1,106  1,106  1,106  1,106 | 0.00  0.10  0.17  0.03 | 0.968  0.752  0.682  0.865 | 126.23  126.13  126.05  126.20 |
| **Soil Type**^2^  Soil Type (Alluvial) 1  (Brown) 2  (Mineral) 3  (Peaty) 4  Alluvial Y  N  ***Brown*** Y  N  Peat Y  N  ***Mineral*** Y  N  Mineral gleys Y  N  ***Mineral podzols*** Y  N  ***Humus-iron podzols (HIP)*** Y  N | 3  37  23  15  3  80  37  46  5  78  38  45  23  60  15  68  20  63 | 1  7  8  8  1  24  17  18  1  24  16  9  8  17  8  17  9  16 | 4,103  1,106  1,106  1,106  1,106  1,106  1,106  1,106 | 0.80  0.01  2.14  0.15  2.49  0.17  2.16  1.37 | 0.526  0.929  ***0.147***  0.701  ***0.117***  0.679  ***0.142***  ***0.245*** | 137.00  126.23  123.97  126.07  123.66  126.06  124.15  124.90 |

1. ***intI2***

| **VARIABLE** | **n*** | | **DF*** | **F*** | ***P**** | **BIC*** |
| --- | --- | --- | --- | --- | --- | --- |
|  | ***Intl2*+**  **(n=89)** | ***Intl2*-**  **(n=19)** |  |  |  |  |
| Animal Health District  (Central) 1  (Highlands) 2  (Islands) 3  (North East) 4  (South East) 5  (South West) 6 | 12  15  15  15  14  18 | 4  3  1  4  3  4 | 5,102 | 0.40 | 0.850 | 126.05 |
| ***Season***  (Autumn) 1  (Winter) 2  (Spring) 3  (Summer) 4 | 25  22  26  16 | 8  2  2  7 | 3,104 | 2.12 | ***0.103*** | 111.73 |
| Herd type  Dairy Y  N  Suckler beef Y  N  Special finisher Y  N | 10  79  60  29  11  78 | 13  10  11  8  3  16 | 1,106  1,106  1,106 | 0.30  0.63  0.16 | 0.553  0.431  0.688 | 109.55  109.22  109.68 |
| Other animals on farm  ***Sheep* Y**  **N**  Wildfowl Y  N  Poultry Y  N  Wild geese Y  N  Gulls Y  N  Breeding bulls Y  N  Foster calves Y  N | 63  26  10  79  24  65  39  50  30  59  26  63  10  79 | 8  11  1  18  5  14  9  10  6  13  5  14  2  17 | 1,106  1,106  1,106  1,106  1,106  1,106  1,106 | 5.36  0.58  0.00  0.08  0.03  0.06  0.01 | ***0.023***  0.448  0.954  0.778  0.859  0.8005  0.929 | 104.39  109.13  109.84  109.76  109.81  109.77  109.83 |
| Water supply  Cattle: mains Y  N  Cattle: spring Y  N  Cattle: natural Y  N  Cattle: natural crosses another farm Y  N  Farmhouse: mains Y  N  Farmhouse: private Y  N | 57  32  45  44  61  28  39  50  65  24  26  63 | 12  7  9  10  11  8  8  11  13  6  6  13 | 1,106  1,106  1,106  1,106  1,106  1,106 | 0.01  0.06  0.79  0.02  0.17  0.04 | 0.942  0.801  0.376  0.891  0.685  0.838 | 109.83  109.77  109.06  109.82  109.68  109.80 |
| Cattle health  Cattle health Y  N  ***Cattle treatment* Y**  **N** | 4  85  28  61 | 3  16  10  9 | 1,106  1,106 | 2.91  2.97 | 0.091  ***0.088*** | 107.21  106.88 |
| Management - Other  ***Brought on other cattle* Y**  **N**  Always isolate Y  N  ***Brought on livestock other than cattle* Y**  **N**  Livestock not owned on farm Y  N  Manure Y  N  Slurry Y  N  ***Cattle housed at sampling* Y**  **N**  Cattle housed with cattle < 1 year old Y  N  Certified Organic Y  N  Change feed Y  N  Change location Y  N  No calving Y  N  Calve all year Y  N | 72  17  47  42  51  38  16  73  53  36  39  50  80  9  37  52  4  85  21  68  16  73  10  79  13  76 | 13  6  10  9  7  12  3  16  13  6  7  12  3  16  0  19  1  18  4  15  3  16  4  15  3  16 | 1,106  1,106  1,106  1,106  1,106  1,106  1,106  -  1,106  1,106  1,106  1,106  1,106 | 1.42  0.00  2.55  0.05  0.51  0.31  28.7  -  0.02  0.06  0.05  1.29  0.02 | ***0.236***  0.989  ***0.113***  0.821  0.475  0.579  ***<0.001***  -  0.885  0.812  0.821  0.258  0.896 | 108.49  109.84  107.19  109.79  109.31  109.52  67.85  -  109.82  109.78  109.79  108.64  109.82 |
| Land Type^1^  ***Arable land Y***  ***N***  ***Mixed Agriculture* Y**  **N**  Improved grassland Y  N  Rough grazing land Y  N | 13  77  57  25  9  80  4  85 | 4  14  18  8  2  17  1  18 | 1,106  1,106  1,106  1,106 | 1.87  1.42  0.02  0.02 | ***0.174***  ***0.236***  0.885  0.885 | 108.10  108.45  109.82  109.82 |
| Soil Type^2^  Soil Type (Alluvial) 1  (Brown) 2  (Mineral) 3  (Peaty) 4  Alluvial Y  N  Brown Y  N  Peat Y  N  Mineral Y  N  Mineral gleys Y  N  Mineral podzols Y  N  Humus-iron podzols (HIP) Y  N | 3  35  46  5  3  86  35  54  5  84  46  43  27  62  19  70  22  67 | 1  9  8  1  1  18  9  10  1  18  8  11  4  15  4  15  7  12 | 4,103  1,106  1,106  1,106  1,106  1,106  1,106  1,106 | 0.22  0.15  0.42  0.00  0.57  0.65  0.00  1.15 | 0.930  0.695  0.520  0.951  0.452  0.422  0.977  0.286 | 123.00  109.70  109.42  109.83  109.26  109.15  109.84  108.73 |

*n, number of herds; DF, degrees of freedom (numerator DF, denominator DF); F, F-statistic; P, *P*-value; BIC, Bayesian Information Criterion.

^1^According to the Macaulay Land Capability for Agriculture classification code, James Hutton Institute, Scotland, UK (<https://www.hutton.ac.uk/learning/exploringscotland/land-capability-agriculture-scotland>)

^2^General soil type defined by James Hutton Institute, Scotland, UK (https://www.hutton.ac.uk/learning/soilshutton/soil-classification)

**Table S8.** One-way analysis of variance^1^ for the *intI1* and *intI2* gene minimum C_t_ value^2^ recorded in a herd, for variables found to be significant at the level of *P* < 0.05 in the univariable models, showing the mean of the herd minimum C_t_ value by variable, with 95% confidence intervals (95% CI).

| **Response** | **Variable (n herds)** | **Mean of**  **Herd minimum C_t_** | **95% CI**  **Herd minimum C_t_** | ***P*-value** |
| --- | --- | --- | --- | --- |
| ***intI1*** | **Animal Health District** | | | **0.050** |
|  | Central (16) | 30.8 | 29.3 – 32.4 |  |
|  | Highland (18) | 31.2 | 29.7 – 32.6 |  |
|  | Islands (16) | 29.5 | 28.0 – 31.1 |  |
|  | North East (19) | 28.3 | 26.9 – 29.8 |  |
|  | South East (17) | 28.6 | 27.1 – 30.2 |  |
|  | South West (22) | 29.5 | 28.1 – 30.8 |  |
|  | **Cattle brought into herd** | | | **0.124** |
|  | Yes (85) | 29.4 | 28.7 – 30.1 |  |
|  | No (23) | 30.6 | 29.2 – 31.9 |  |
|  | **Wild geese on fields** | | | **0.069** |
|  | Yes (48) | 30.3 | 29.3 – 31.2 |  |
|  | No (60) | 29.1 | 28.3 – 29.9 |  |
|  | **Cattle spring water** | | | **0.025** |
|  | Yes (54) | 28.9 | 28.1 – 29.8 |  |
|  | No (54) | 30.3 | 29.5 – 31.2 |  |
|  | **Group housed at sampling** | | | **0.016** |
|  | Yes (83) | 29.2 | 28.5 – 29.9 |  |
|  | No (25) | 31.0 | 29.7 – 32.3 |  |
|  | **Number of cattle 12 to 30 months^3^** | Pearson correlation, -0.196 | | **0.042** |
| ***intI2*** | **Season** | | | **0.153** |
|  | Autumn (33) | 29.9 | 28.6 – 31.2 |  |
|  | Winter (24) | 32.1 | 30.7 – 33.5 |  |
|  | Spring (28) | 31.0 | 29.8 – 32.2 |  |
|  | Summer (23) | 30.5 | 29.1 – 31.9 |  |
|  | **Sheep present** | | | **0.003** |
|  | Yes (71) | 30.1 | 29.4 – 30.9 |  |
|  | No (37) | 32.2 | 31.1 – 33.3 |  |
|  | **Group housed at sampling** | | | **<0.001** |
|  | Yes (83) | 29.7 | 29.1 – 30.3 |  |
|  | No (25) | 34.6 | 33.5 – 35.7 |  |

^1^One way analysis of variance and Pearson correlation performed in Minitab, Version 18.1

^2^The minimum C_t_ value recorded in a herd, from all available pools tested; for herds where no trace was recorded in any pool due to negativity, the minimum C_t_ value was set to the PCR cycle limit number, C_t_=40.

^3^(Log_10_) transformed

**Figure S6.** The mean minimum recorded herd C_t_ value for *intI1* and *intI2* in grazed cattle (G) compared to housed cattle (H), showing 95% CI of the mean.
